# ODR-1 acts in AWB neurons to determine the sexual identity of *C. elegans* pheromone blends

**DOI:** 10.17912/micropub.biology.000507

**Published:** 2022-01-13

**Authors:** Erin Z. Aprison, Ilya Ruvinsky

**Affiliations:** 1 Department of Molecular Biosciences, Northwestern University, Evanston, IL 60208, USA

## Abstract

Valence of animal pheromone blends can vary due to differences in relative abundance of individual components. For example, in *C. elegans*, whether a pheromone blend is perceived as “male” or “hermaphrodite” is determined by the ratio of concentrations of ascr#10 and ascr#3. The neuronal mechanisms that evaluate this ratio are not currently understood. We present data that suggest that the function of guanylyl cyclase ODR-1 in AWB neurons is required for the effect of ascr#3 that counteracts the activity of ascr#10. This finding defines a new module in the neuronal mechanism that determines the sexual identity of *C. elegans* pheromone.

**Figure 1. Germline response to ascr#10 and ascr#3 in hermaphrodites carrying mutations that affect subsets of the nervous system:**
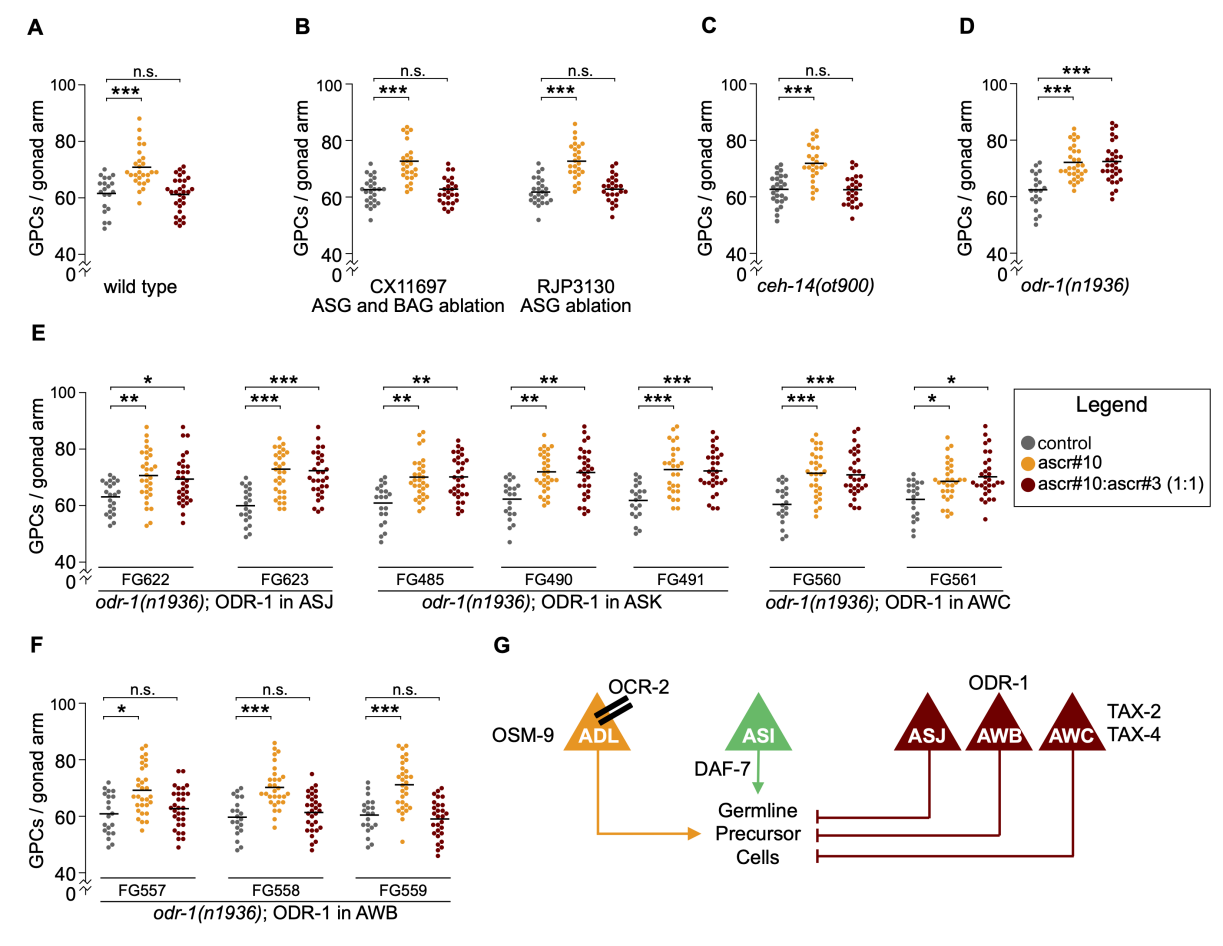
In panels A-F, each dot represents the number of GPCs in one gonad arm of one hermaphrodite; grey dots represent animals raised on control plates, orange dots represent animals exposed to ascr#10 starting at 48 hours after release from L1 arrest (after the L4/adult transition but before the onset of egg laying) until being tested on Day 5 of adulthood, red dots represent animals that were exposed to a 50:50 blend of ascr#10 + ascr#3. (A) The germline response to ascr#10 and a blend of ascr#10 + ascr#3 in wild type N2 hermaphrodites. This panel illustrates the logic of our paradigm – exposure to ascr#10 increases the number of germline precursor cells (GPCs), whereas exposure to a 50:50 blend of ascr#10 + ascr#3 has no effect. Inability to respond to ascr#3 would result in a similar increase in GPCs on ascr#10 as on the ascr#10 + ascr#3 blend. Data in this panel were previously published (Aprison and Ruvinsky, 2017). The germline response to ascaroside pheromones in (B) two strains that ablate ASG and BAG neurons, (C) *ceh-14(ot900)*,(D) *odr-1(n1936)*, (E) *odr-1(n1936)* that express wild type ODR-1 in ASJ, ASK, AWC neurons, (F) *odr-1(n1936)* that express wild type ODR-1 in AWB neurons. In panel A, N=20 (control), N=27 (ascr#10), and N=30 (ascr#10 + ascr#3). In panels B-F, N=25 for each condition. In panels A-F, horizontal black lines mark the mean. Asterisks indicate statistical significance as determined by the Kolmogorov-Smirnov test (*p<0.05, ** p<0.01, and ***p<0.001). (G) A model of sensory neurons and pathways required for the germline response to ascaroside pheromones. ADL and ASI are required to increase the number of GPCs in response to ascr#10. ASJ, AWB, and AWC mediate the antagonizing effects of ascr#3. Modified from (Aprison and Ruvinsky, 2017).

## Description

In *C. elegans*, complex blends of small molecules act as pheromones; the best known of these are ascarosides (Srinivasan *et al.*, 2008; Srinivasan *et al.*, 2012). Although hermaphrodites and males produce similar ascaroside profiles, there are several notable and functionally consequential differences. The best studied one is that males excrete blends enriched in ascr#10, whereas hermaphrodite blends are enriched in a nearly-identical (a difference of a single unsaturated bond) ascr#3 (Izrayelit *et al.*, 2012). Previously, we showed that hermaphrodites exposed to the male pheromone have an enlarged population of germline precursor cells (Aprison and Ruvinsky, 2016). This effect can be recapitulated by physiological concentrations of synthetic ascr#10 and ascr#3 (Aprison and Ruvinsky, 2016), as long as the concentration of the male-enriched ascr#10 is higher than that of ascr#3 (Aprison and Ruvinsky, 2017). Central to discriminating between blends with different concentration ratios of these two ascarosides is that ascr#3 counteracts the effect of ascr#10, resulting in no discernable effect when the concentration of ascr#3 was greater or equal to that of ascr#10 (Aprison and Ruvinsky, 2017). Therefore, in our paradigm, a mutant strain can be tested for the ability to respond to ascr#3 only if it responds to ascr#10 (this response is manifested as an increased number of germline precursors). In the absence of ascr#10 response, ascr#3 response cannot be assessed because this compound does not change the number of germline precursors (Figure 1A).

Using this approach, we identified a set of six pairs of sensory neurons (ASG, ASI, ASJ, ASK, AWB, and AWC), at least some of which being required for the ascr#3 response (Aprison and Ruvinsky, 2017). Our previous experiments with neuron-specific ablations established that ASJ, AWB, and AWC neurons were required for ascr#3 response, whereas ASI were required for ascr#10 response, and ASK played no obvious role in our paradigm (Aprison and Ruvinsky, 2017). In the present study, using strains that ablate ASG (as well as BAG) neurons (Juozaityte *et al.*, 2017), we found that loss of these cells does not affect hermaphrodites’ ability to respond to ascr#3 (Figure 1B). Similarly, mutants carrying a strong loss-of-function allele in a LIM homeobox gene *ceh-14(ot900)* (Bayer and Hobert, 2018) had a wild type ascr#3 response (Figure 1C). These results argue that response to ascr#3 does not require ASG, BAG, and ~20 neurons that express *ceh-14* (Bayer and Hobert, 2018; Cassata *et al.*, 2000; Kagoshima *et al.*, 2013; Taylor *et al.*, 2021).

The six pairs of sensory neurons, ASG, ASI, ASJ, ASK, AWB, and AWC, express *tax-2* and *tax-4* genes encoding subunits of a cyclic nucleotide-gated channel. These cells also express *odr-1* (Taylor *et al.*, 2021), a gene that encodes a receptor guanylate cyclase thought to supply cGMP to the TAX-2/TAX-4 channel (L’Etoile and Bargmann, 2000). Loss of *odr-1* eliminated ascr#3 response (Figure 1D). To identify the site of *odr-1* action, we used strains that narrowly expressed the wild type ODR-1 protein in the background of the loss-of-function mutant *odr-1(n1936)* (Krzyzanowski *et al.*, 2016). We focused on the latter four neuronal pairs because loss of ASG does not affect ascr#3 response (Figure 1B), while ASI is involved in ascr#10 response and therefore could not be tested in this paradigm (Aprison and Ruvinsky, 2017). Expression of ODR-1 under control of *trx-1* (in ASJ), *srbc-66* (inASK), or *ceh-36* (in AWC) did not rescue the *odr-1(n1936)* mutant phenotype (Figure 1E). A comprehensive gene expression atlas detected transcripts of *trx-1*, *srbc-66,* and *ceh-36* in few additional cells (PVR, AWA, and ASE, respectively); these neurons do not appear to express *odr-1* (Taylor *et al.*, 2021). Expression of ODR-1 under control of *str-1* robustly rescued *odr-1(n1936)* (Figure 1F). *str-1* transcripts were present in AWB and RIP neurons, two neuronal classes in which *odr-1* was also detected (Taylor *et al.*, 2021). Because no ODR-1 function in RIP has been described, whereas AWB is a major site of ODR-1 activity (L’Etoile and Bargmann, 2000), we currently favor the hypothesis that, with respect to response to ascr#3, ODR-1 acts in AWB neurons.

It is not currently clear whether ODR-1 in AWB is involved in sensing ascr#3. Although AWB are amphid sensory neurons, they are generally thought to sense volatile repellents (Bargmann, 2006), whereas ascarosides do not appear to be volatile. It is possible that ODR-1 in AWB modulates activity of an ascr#3-sensing neuron(s) or is required further downstream for the germline response to ascr#3. Because our paradigm relied on scoring the population of germline precursor cells, we conservatively conclude that all neurons and gene products identified so far are involved in “the germline response to ascr#10 and ascr#3”, although we assume that at least some of these are required for sensing the two sex pheromones and comparing their concentrations. The current state of knowledge of the sensory components involved in the germline response to ascarosides is summarized in Figure 1G.

## Methods

We used standard *C. elegans* methods as previously published (Aprison and Ruvinsky, 2017, 2019a, b). Additional protocol details are available upon request.

## Reagents

**Table:** 

**Strain**	**Genotype**	**Available from**
N2	wild type	CGC
CX11697	*kyIs536[flp-17::p17::SL2::GFP, elt-2::mCherry]*	Bargmann lab
RJP3130	*otEx3900 [ops-1p::*p12*; gcy-21p::*p17*; myo-3p::RFP]; rpEx1523[ets-5p::mCherry; elt-2p::GFP]*	Pocock lab
OH15422	*ceh-14(ot900)*	CGC
CX2065	*odr-1(n1936)*	CGC
FG622	*odr-1(n1936); udEx232[trx-1p::odr-1; elt-2p::gfp]*	Ferkey lab
FG623	*odr-1(n1936); udEx231[trx-1p::odr-1; elt-2p::gfp]*	Ferkey lab
FG485	*odr-1(n1936); udEx276[srbc-66p::odr-1; elt-2p::gfp]*	Ferkey lab
FG490	*odr-1(n1936); udEx214[srbc-66p::odr-1; elt-2p::gfp]*	Ferkey lab
FG491	*odr-1(n1936); udEx215[srbc-66p::odr-1; elt-2p::gfp]*	Ferkey lab
FG560	*odr-1(n1936); udEx307[ceh-36p3::odr-1; elt-2p::gfp]*	Ferkey lab
FG561	*odr-1(n1936); udEx308[ceh-36p3::odr-1; elt-2p::gfp]*	Ferkey lab
FG557	*odr-1(n1936); udEx304[str-1p::odr-1; elt-2p::gfp]*	Ferkey lab
FG558	*odr-1(n1936); udEx305[str-1p::odr-1; elt-2p::gfp]*	Ferkey lab
FG559	*odr-1(n1936); udEx306[str-1p::odr-1; elt-2p::gfp]*	Ferkey lab
